# Roles of mitochondrial complexes in non-alcoholic fatty liver disease

**DOI:** 10.3389/fmolb.2026.1752024

**Published:** 2026-02-23

**Authors:** Kai Wang, Li Wang, Jiamin Ning, Dexin Li

**Affiliations:** 1 Rehabilitation Medicine Department of The Second Affiliated Hospital, School of Medicine, The Chinese University of Hong Kong, Shenzhen, & Longgang District People's Hospital of Shenzhen, Shenzhen, Guangdong, China; 2 Department of Radiotherapy, The Second Hospital of Chaoyang (Women’s and Children’s Hospital of Chaoyang), Chaoyang, Liaoning, China; 3 School of Public Health, Jilin University, Changchun, China; 4 Department of Anesthesia, The Second Clinical Medical College, Xi’an Medical College, Xi’an, Shaanxi, China; 5 School of Basic Medical Sciences, Peking University, Beijing, China

**Keywords:** energy metabolism, lipid metabolism, mitochondrial complexes, non-alcoholic fatty liver disease (NAFLD), redox homeostasis

## Abstract

Non-alcoholic fatty liver disease (NAFLD) is increasingly recognized as a mitochondrial-driven metabolic disorder, yet the specific contributions of individual mitochondrial respiratory chain complexes remain poorly defined. In particular, inconsistent alterations in complexes I–V have been reported across different NAFLD models, representing a critical knowledge gap. Here, we systematically reviewed *in vivo* and *in vitro* studies to evaluate changes in mitochondrial complexes I–V during NAFLD progression. Overall, NAFLD is commonly associated with reduced complex activity, impaired mitochondrial respiration, and increased reactive oxygen species production. Notably, a subset of studies reported enhanced complex activity and respiration, suggesting context-dependent mitochondrial adaptations. This synthesis clarifies divergent findings and highlights mitochondrial respiratory complexes as dynamic and therapeutically relevant targets for future NAFLD intervention strategies.

## Graphical Abstract

**Figure:**
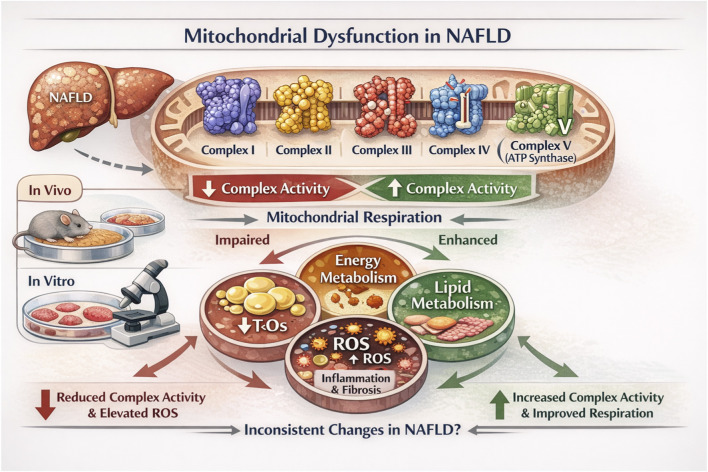

## Introduction

Non-alcoholic fatty liver disease (NAFLD) is a metabolic liver disorder characterized by excessive hepatic lipid accumulation that can progress from simple steatosis to steatohepatitis, fibrosis, and hepatocellular carcinoma (Wei et al., 2024; Tian et al., 2024; Tang, 1990). Increasing evidence indicates that mitochondrial dysfunction, particularly alterations in the mitochondrial respiratory chain, plays a central role in NAFLD pathogenesis (Xu et al., 2025a; Xu et al., 2025b; Wedan et al., 2024). As the core machinery for oxidative phosphorylation, mitochondrial complexes I–V are essential for hepatic energy production and lipid metabolic homeostasis. Structural or functional impairment of these complexes disrupts fatty acid oxidation and ATP synthesis, leading to lipid droplet accumulation and excessive reactive oxygen species (ROS) (Mercola, 2025; Zheng et al., 2023). The resulting oxidative stress, inflammation, and hepatocyte injury provide a mechanistic basis for disease progression, highlighting mitochondrial respiratory chain complexes as critical determinants and potential therapeutic targets in NAFLD (Wei et al., 2025).

Mitochondrial complexes play a crucial role in NAFLD, as they are involved not only in energy metabolism and lipid metabolism but also in oxidative stress, inflammatory responses, and liver fibrosis, among other pathological processes (Mu et al., 2025). Complexes I–V, as essential components of the mitochondrial electron transport chain, are vital for maintaining cellular function. Functional impairment of these complexes restricts ATP synthesis while enhancing reactive oxygen species production, thereby triggering cellular injury, metabolic imbalance, and progressive hepatic dysfunction, which collectively accelerate NAFLD onset and progression (Li et al., 2025). Furthermore, recent studies have revealed that the activity of mitochondrial complexes is regulated by various endogenous factors, which may serve as potential intervention targets (Juárez-Fernández et al., 2023).

Therefore, a systematic study of the structural and functional changes of mitochondrial complexes, as well as their roles at different stages of NAFLD, will help to deepen our understanding of the pathogenesis of NAFLD and provide a theoretical foundation and practical direction for developing new therapeutic strategies and identifying effective treatment targets. In this regard, this review summarizes the research progress on mitochondrial complexes I–V in non-alcoholic fatty liver disease and their potential regulatory mechanisms.

### Literature search strategy and inclusion criteria

This review focuses on the role of mitochondrial respiratory chain complexes in non-alcoholic fatty liver disease (NAFLD) and was based on a systematic search of the PubMed database. Search terms included “mitochondrial complex I–V″ combined with “non-alcoholic fatty liver disease” or “NAFLD” together with relevant synonyms and MeSH terms. Priority was given to original research articles published within the past 5 years in high–impact factor journals, with inclusion requiring explicit evaluation of changes in the activity, expression, or function of mitochondrial respiratory chain complexes. The search results indicated that studies related to complex I were the most abundant (see Table 1), whereas reports on complexes IV and V were relatively limited; therefore, complexes IV and V were discussed together based on their lower representation and mechanistic similarities (see Table 4).

**TABLE 1:** Specific association between mitochondrial complex I dysfunction and NAFLD.

Model	Mitochondrial complex I target	Biological effect	References
Choline Deficiency High-Fat Diet (CDAHFD) Mice	NDUFS2 and NDUFA10	Decreased expression of NDUFS2 and NDUFA10, leading to reduced Complex I enzyme activity	Piekutowska-Abramczuk et al. (2018)
Obese Patients with Liver Steatosis (human)	NDUFB8 and NDUFB9	Reduced protein levels of NDUFB8 and NDUFB9	Li et al. (2021)
Parenteral Nutrition-related Liver Disease Model	NDUFS1	Overexpression of NDUFS1 alleviates oxidative stress	Wan et al. (2023)
Fat Emulsion-induced L02 Hepatocyte Model (human)	Overall Complex I	Reduced mitochondrial Complex I and II content	Mu et al. (2021)
High-fat High-cholesterol Diet Rat Model	Overall Complex I	Reduced activity of mitochondrial Complex I and II	Sangineto et al. (2021)
High-fat Diet Induced NAFLD Rat Model	Overall Complex I	Reduced Complex I activity	Yang et al. (2019)
Cholesterol Diet-induced Mouse Model	Overall Complex I	Reduced respiration of Complex I and II; decreased supercomplex (I1 + III2 + IV) and (I1 + III2)	Solsona-Vilarrasa et al. (2019)
High-fat Diet-fed Rat Model	Overall Complex I	Reduced Complex I activity	García-Berumen et al. (2019)
HFD-induced Obese Mouse Model	Overall Complex I	Reduced activity of mitochondrial Complex I, IV, and V	Khoo et al. (2019)
High-fat Diet-fed Mouse Model	Overall Complex I	Reduced activity of mitochondrial Complex I, II, and IV	Trzepizur et al. (2015)
High-fat/High-sucrose Diet-fed Rat Model	Overall Complex I	Reduced activity of mitochondrial Complex I and II	Chu et al. (2015)
High-fat High-sucrose Diet-fed Mouse	Overall Complex I	Reduced activity of Complex I and IV, ATP depletion	Verbeek et al. (2015)
High-fat Diet-fed Mouse Model	Overall Complex I	Reduced Complex I respiration	Kučera et al. (2014)
Choline Deficiency Diet-fed Rat Model	Overall Complex I	Decreased Complex I in mitochondria	Petrosillo et al. (2007)
Free Fatty Acid (FFA) treated HepaRG Cells (human)	Overall Complex I	Increased activity of Complex I and II	Maseko et al. (2024)
High-fat Diet (HFD) Fed Mouse	Overall Complex I	Increased Complex I respiration with no significant effect on Complex II and IV	Yu et al. (2021)
High-fat Diet-fed Mouse Model	Overall Complex I	Slight increase in expression of Complex I and III, enhanced mitochondrial respiration	Sun et al. (2017)

### The specific link between mitochondrial complex dysfunction and NAFLD

#### The link between complex I dysfunction and NAFLD

Mitochondrial complex I (NADH dehydrogenase, NADH:ubiquinone oxidoreductase), consisting of 45 subunits, is one of the largest known proteins, with a molecular weight of 980 kDa (Fiedorczuk and Sazanov, 2018). Complex I is located in the inner mitochondrial membrane and transfers electrons from NADH to CoQ while coupling the ejection of four protons from the mitochondrial matrix to the intermembrane space, generating a proton gradient across the membrane that drives ATP synthesis (Laube et al., 2024). Recent studies have shown that mitochondrial complex I also plays a critical role in sodium ion transport, thereby contributing to the formation of the mitochondrial membrane potential (Hernansanz-Agustín et al., 2024).

The references included in this review employed multiple experimental approaches to evaluate the function and status of mitochondrial respiratory chain complexes. Among these, enzyme activity assays, Seahorse analysis, and blue native polyacrylamide gel electrophoresis (BN-PAGE) reflect distinct aspects of mitochondrial complex properties and are therefore highly complementary (Jha et al., 2016; Arroum et al., 2023). Enzyme activity assays are typically performed using isolated mitochondria or tissue homogenates and provide a direct assessment of the catalytic activity of individual respiratory chain complexes, but they are limited in their ability to capture the functional contribution of these complexes to integrated cellular respiration (Wang et al., 2025). Seahorse analysis assesses global mitochondrial respiratory function and metabolic flexibility by real-time measurement of oxygen consumption and glycolytic activity, thereby reflecting the overall impact of complex dysfunction on cellular energy metabolism, while lacking resolution of the structural status of specific complexes (Desousa et al., 2023). In contrast, BN-PAGE is primarily used to analyze the assembly, organization, and stability of mitochondrial respiratory chain complexes and supercomplexes, revealing structural alterations, but does not directly measure enzymatic activity or dynamic respiratory function. Accordingly, each experimental approach provides distinct and complementary information.

Mitochondrial complex I is one of the most extensively studied complexes in mitochondrial research (Agip et al., 2019). A range of factors can modulate its activity, and alterations in complex I function contribute to mitochondrial dysfunction, thereby playing a critical role in the pathogenesis of NAFLD (Ding et al., 2022). The below summarizes the specific link between mitochondrial complex I dysfunction and NAFLD (Table 1). Most studies (Solsona-Vilarrasa et al., 2019; García-Berumen et al., 2019; Verbeek et al., 2015) indicate that high-fat, high-cholesterol, high-sugar, and choline-deficient diets induced NAFLD, which is associated with decreased complex I activity, leading to mitochondrial defects, suppressed respiration, increased lactate dehydrogenase (LDH) activity, ATP depletion, a reduced respiratory control ratio, and increased ROS production. However, some studies (Maseko et al., 2024; Yu et al., 2021; Sun et al., 2017) have also reported an increase or slight increase in complex I activity and expression in NAFLD models, leading to enhanced mitochondrial respiration and the generation of more ROS, which causes oxidative stress. Whether complex I activity is increased or decreased, mitochondrial dysfunction and oxidative damage are evident, which are key factors in the onset and progression of NAFLD.

In research on mitochondrial complexes and NAFLD, several key subunits of mitochondrial complex I play critical roles. NDUFS1 (NADH: ubiquinone oxidoreductase core subunit S1), the largest core subunit of complex I, can modulate the activity of the mitochondrial respiratory chain complex I and the formation of mitochondrial ROS (Qi et al., 2022). NDUFS1 participates in electron transfer from NADH to coenzyme Q and is functionally coupled to proton translocation from the mitochondrial matrix to the intermembrane space, a process that is essential for preserving the structural integrity and proper function of complex I (Ni et al., 2019). Its overexpression promotes the formation of supercomplexes by complex I, effectively reducing the production of reactive oxygen species (ROS). This NDUFS1-mediated assembly of complex I into supercomplexes plays an important role in regulating ROS production and oxidative stress (Lopez-Fabuel et al., 2016). In patients with non-alcoholic fatty liver disease (NAFLD), the expression of NDUFS1 is reduced. Overexpression of NDUFS1 can reduce complex I activity and alleviate the progression of NAFLD (Wan et al., 2023). NDUFS2 (NADH: ubiquinone oxidoreductase core subunit S2) and NDUFA10 (NADH: ubiquinone oxidoreductase core subunit A10) are key components of mitochondrial respiratory complex I (Ding et al., 2022). NDUFS2 is located in the Q module 27, while NDUFA10 resides in the hydrophobic protein ND2 module of complex I. Both are involved in the assembly of complex I. The enzymatic activity of complex I is a rate-limiting step in oxidative phosphorylation and is an important regulatory factor. When complex I activity is reduced, it blocks electron transfer in the respiratory chain, leading to an imbalance between electron input and output (Dunham-Snary et al., 2019; Hoefs et al., 2011). This imbalance stimulates ROS production, which can potentially trigger severe liver disease (Ding et al., 2022). The NDUFB8 subunit of NADH:ubiquinone oxidoreductase contributes to the assembly of mitochondrial respiratory complex I, with this process occurring in both the endoplasmic reticulum and mitochondria (Piekutowska-Abramczuk et al., 2018). The NDUFB9 subunit of NADH: ubiquinone oxidoreductase B9 is localized to the mitochondrial inner membrane and is responsible for dehydrogenating nicotinamide adenine dinucleotide (NADH) and transferring electrons to coenzyme Q (Wu et al., 2016). NDUFB9 plays a key role in the oxidative phosphorylation reaction and is involved in the electron transfer process of the mitochondrial respiratory chain. Studies have shown that during the lipogenesis process of NAFLD, NDUFB9 expression is significantly upregulated, and silencing NDUFB9 (with an 83% silencing efficiency) inhibits lipogenesis (Zhu et al., 2022). As part of complex I, NDUFB8 and NDUFB9 may serve as biomarkers reflecting mitochondrial status, playing a role in the diagnosis and monitoring of NAFLD (Piekutowska-Abramczuk et al., 2018; Li et al., 2015).

In addition, it is important to note the differences among commonly used dietary NAFLD models (Ning et al., 2025; Tsuchida et al., 2018). As summarized in the tables of this review, HFD and WD models primarily mimic metabolic syndrome–associated NAFLD, characterized by pronounced obesity and insulin resistance, with only mild impairment of mitochondrial complexes and potential compensatory enhancement. In contrast, CDAHFD and MCD models rapidly induce severe steatohepatitis and fibrosis, though their metabolic contexts differ (MCD does not cause obesity or insulin resistance) (Yang, 2024; Suzuki-Kemuriyama et al., 2021). Mitochondrial complexes are markedly impaired in these models, with decreased ATP production and increased ROS generation. The differential effects of these models on complexes I–V help explain the variability in mitochondrial activity reported across studies.

Changes in mitochondrial complex activity affect the onset and progression of NAFLD, and the factors influencing complex I function, as described above, offer insights into the prevention and treatment of NAFLD. Most current studies using Seahorse focus on the overall activity of mitochondrial complexes I and II, but there is limited research on how specific subunits and the interactions between subunits impact the function of mitochondrial complex I.

#### The link between complex II dysfunction and NAFLD

Mitochondrial complex II (Complex II), also known as succinate dehydrogenase (SDH), is an important functional complex in the mitochondrial electron transport chain (Guo et al., 2017). It connects two essential physiological metabolic processes, the tricarboxylic acid cycle (TCA) and oxidative phosphorylation (OXPHOS) (Sharma et al., 2020). Complex II is a transmembrane protein complex that plays a key role in cellular respiration. It is responsible for oxidizing succinate to fumarate and transferring electrons to ubiquinone (coenzyme Q), thus participating in the electron transport chain (Sharma et al., 2024). In mammals, the electron transport chain (ETC) complexes I, III, and IV contain 45, 11, and 13 subunits, respectively (Zhu et al., 2016), composed of proteins encoded by both the mitochondrial and nuclear genomes. In contrast, complex II (SDH) is composed of only four nuclear-encoded subunits: SDHA, SDHB, SDHC, and SDHD. The SDHA subunit contains covalently bound flavin adenine dinucleotide (FAD) and is responsible for removing electrons from succinate (Sharma et al., 2024). The SDHB subunit contains three iron-sulfur clusters, which mediate the transfer of electrons to the ubiquinone binding site (Q site) on ubiquinone. The SDHC and SDHD subunits are embedded in the mitochondrial inner membrane, anchoring the entire complex to the membrane (Sharma et al., 2020; Wang et al., 2023).

Succinate accumulation exerts a dual role in inflammation (Myint et al., 2023): it functions both as a metabolic intermediate and as an immunological signaling molecule, promoting pro-inflammatory responses through HIF-1α stabilization, NLRP3 inflammasome activation, SUCNR1 signaling, and mitochondrial ROS production, particularly in M1 macrophages and acute inflammatory contexts (Huang et al., 2024; Chen et al., 2024). Moreover, when succinate feeds electrons into the mitochondrial electron transport chain via succinate dehydrogenase (SDH), reverse electron transport (RET) can occur to Complex I under high membrane potential, further enhancing ROS generation (Schönfeld et al., 2010; Schönfeld and Wojtczak, 2007). These ROS not only induce oxidative stress but also amplify the secretion of pro-inflammatory cytokines such as IL-1β, TNF-α, and IL-6 via the aforementioned pathways, driving metabolic reprogramming and exacerbating inflammatory responses (Keiran et al., 2019; Gobelli et al., 2023).

The primary function of mitochondrial complex II is to catalyze the oxidation of succinate to fumarate, while directly transferring electrons to the ubiquinone pool (Goetz et al., 2023). This process is crucial for maintaining the integrity of the mitochondrial electron transport chain and cellular energy metabolism. Additionally, complex II is involved in regulating the cell’s metabolic and respiratory adaptation to various intrinsic and extrinsic stimuli, influencing mitochondrial function and cellular energy metabolism (Iverson et al., 2023). Mitochondrial complex II plays a central role in cellular energy metabolism and mitochondrial function, and abnormalities in its structure and function are associated with various diseases (Hadrava Vanova et al., 2020). SDH creates the unique direct functional link between the tricarboxylic acid cycle (TCA) and oxidative phosphorylation (OXPHOS), and it is ideally positioned to coordinate flux through both pathways. Unlike complex I, the transfer of electrons from SDH to the ubiquinone pool is not limited by the high proton gradient (Yu et al., 2023).

The table below summarizes the specific link between mitochondrial complex II dysfunction and NAFLD (Table 2). Researchers have found that in NAFLD, the expression of the SDHA and SDHB genes is reduced, and SDH activity is decreased, while protein expression of SDH subunits is increased (Staňková et al., 2021). However, other studies observed that Western diet (WD) did not significantly affect the gene and protein expression of the SDHA subunit (Staňková et al., 2020). This discrepancy may be related to factors involved in the assembly of subunits into functional complexes, which mediate the stability of intermediates during the assembly of individual subunits and the multimeric complex (Van Vranken et al., 2015). In NAFLD mice, mitochondrial damage resulting from oxidative stress and/or impaired mitochondrial autophagy mechanisms can lead to blockage in the later stages of the TCA cycle and failure in energy conversion (Liu et al., 2020). This may be due to mitochondrial dysfunction, leading to inhibition of mitochondrial SDHA enzyme levels and/or activity, causing succinate accumulation in hepatocytes. These studies highlight the close relationship between mitochondrial complex II and NAFLD. Whether considering the overall complex II or its subunits SDHA and SDHB, they can serve as targets for monitoring and treatment of NAFLD. Both the entire mitochondrial complex II and the SDHA and SDHB subunits have been extensively studied as potential therapeutic targets. Most studies (Staňková et al., 2021; Staňková et al., 2020; Nilsson et al., 2023) show that in NAFLD models, complex II activity is reduced, oxidative phosphorylation levels are decreased, and ketogenesis is enhanced. A few studies (Sangineto et al., 2021; Aghayev et al., 2023), however, show increased complex II activity and enhanced fatty acid oxidation. This discrepancy warrants further in-depth investigation.

**TABLE 2:** Mitochondrial complex II dysfunction and its specific association with NAFLD.

Model	Mitochondrial complex II target	Biological effect	References
Western Diet (WD) Fed Mouse Model	SDHB	Decreased protein expression of Complex II, III, and V in high-fat diet model	Nilsson et al. (2023)
Western Diet Induced Mouse Model	Overall Complex II, SDHA, SDHB	Reduced Complex II activity, decreased SDHA and SDHB gene expression, increased protein expression	Staňková et al. (2021)
Dihydrotestosterone (DHT) Induced NALFD Rat Model	SDHB	Reduced Complex II activity, increased ROS production	Cui et al. (2021)
Western Diet (WD) Induced NAFLD Mouse Model	Overall Complex II, SDHA	Reduced succinate dehydrogenase (SDH) activity, no significant change in SDHA protein expression	Staňková et al. (2020)
High-fat/High-calorie Diet Induced Mouse Model	SDHA	Reduced expression of SDHA gene and protein levels	Liu et al. (2020)
Methionine and Choline Deficient Diet Mouse Model	Overall Complex II, SDHA	Decreased Complex II activity, reduced SDHA protein expression	Fernández-Tussy et al. (2019)
Cholesterol-enriched Diet-fed Mouse Model	Overall Complex II	Reduced respiration of Complex I and Complex II	Solsona-Vilarrasa et al. (2019)
HFD Induced Fatty Degeneration Mouse Model	SDHB	Increased SDHB protein expression	Aghayev et al. (2023)
High-fat/High-cholesterol Diet Induced Rat Model	Overall Complex II	Enhanced Complex II activity, reduced activity of Complex I, III, and V	Sangineto et al. (2021)

Current studies indicate that mitochondrial respiratory chain complex function in high-fat–related NAFLD models can be either enhanced or impaired, resulting in inconsistencies among reported findings (Wang et al., 2025; Tsuchida et al., 2018; Yang, 2024). Overall, this variability can be attributed to several factors. First, differences in modeling strategies play a decisive role: commonly used dietary models—including high-fat diet (HFD), Western diet (WD), choline-deficient high-fat diet (CDAHFD), and methionine- and choline-deficient diet (MCD)—differ markedly in metabolic background and disease severity. HFD and WD primarily recapitulate metabolic syndrome–associated NAFLD, with relatively mild mitochondrial damage and, in some cases, compensatory functional enhancement, whereas CDAHFD and MCD rapidly induce severe steatohepatitis and fibrosis, causing pronounced impairment of complexes I–V (Suzuki-Kemuriyama et al., 2021). Second, discrepancies between *in vivo* and *in vitro* models are significant, as hepatocyte-based *in vitro* systems cannot fully replicate the complex hepatic microenvironment or intercellular mitochondrial interactions present *in vivo*. Third, NAFLD is a dynamic disease, and mitochondrial function may shift from early compensatory enhancement to late-stage decline; however, most studies assess mitochondria at a single time point. Fourth, species-specific differences in mitochondrial regulation and metabolic adaptation further limit the direct comparability of findings across studies (Guo et al., 2022).

#### Complex III, ROS generation, and oxidative stress

Complex III plays a central role in the electron transport chain, where it is primarily responsible for oxidizing ubiquinol generated by complexes I and II (Zheng et al., 2024). This oxidation process is accompanied by the pumping of two protons into the mitochondrial intermembrane space, while electrons are transferred to cytochrome c in the intermembrane space, facilitating electron transfer in the electron transport chain and the reduction of cytochrome c (Letts and Sazanov, 2017). The core structure of complex III is composed of three key protein subunits: cytochrome b, which contains two heme groups, b562/bH and b566/bL. Cytochrome b is a highly hydrophobic transmembrane protein, and its precise role in the electron transport chain is not yet fully understood (Flores-Mireles et al., 2023). Cytochrome c1 contains a heme c1 group. It consists of a larger hydrophilic region and a smaller hydrophobic region. The hydrophilic region carries the heme cofactor and extends into the aqueous phase outside the membrane, where it binds with cytochrome c (Guo et al., 2017). Additionally, there is the iron-sulfur protein (FeS protein), which participates in various redox reactions. Structural and functional abnormalities in FeS proteins may lead to metabolic disorders or dysfunctions. The main function of FeS proteins is to participate in redox reactions, as the iron ions in their cofactors can switch between Fe2+ and Fe3+ states (Lill and Freibert, 2020).

Complex III plays a central role in the mitochondrial electron transport chain through the Q cycle, which mediates electron transfer from ubiquinol to cytochrome c (Murphy, 2009). During this process, the Qo site of Complex III is a major site of mitochondrial ROS generation, particularly when electron flow is impaired or the CoQ pool is over-reduced (Hernansanz-Agustín and Enríquez, 2021). In the context of NAFLD, dysfunction at Complex III can lead to excessive ROS production, contributing to oxidative stress, lipid peroxidation, and inflammatory signaling, thereby exacerbating hepatocellular injury and disease progression. Understanding the Q cycle and ROS generation at the Qo site is therefore critical for elucidating the mechanistic role of Complex III in NAFLD (Moreno-Loshuertos and Enríquez, 2016). UQCRC2, a subunit of mitochondrial respiratory chain complex III, has become a target of several studies (Fernandez-Vizarra and Zeviani, 2018). Among the five articles (García-Berumen et al., 2022; Sarr et al., 2019; Lee et al., 2018; Abdelmegeed et al., 2016; Ronis et al., 2013) related to NAFLD (see Table 3), four report a decrease in the activity of complex III as a whole or its subunits, leading to mitochondrial respiratory dysfunction, followed by increased ROS production and oxidative stress. Another study (Ronis et al., 2013), however, observed an enhancement in complex III activity, which subsequently improved mitochondrial respiration. Given the significant differences in these findings, it is worth further investigating the underlying mechanisms and potential impacts.

**TABLE 3:** Mitochondrial complex III dysfunction and its specific association with NAFLD.

Model	Mitochondrial complex III target	Biological effect	References
High-fat/High-fructose Diet Induced NAFLD Mouse Model	Overall Complex III	Reduced Complex III activity, mitochondrial respiratory dysfunction	García-Berumen et al. (2022)
High Saturated Fat/High Sucrose-Fructose Diet Guinea Pig Model	Complex III	Reduced oxidative phosphorylation Complex III activity	Sarr et al. (2019)
Cholesterol-Enriched High Saturated Fat Diet Induced Mouse Model	Overall Complex III, UQCRC2	Decreased Complex III content, UQCRC2 protein unchanged	Lee et al. (2018)
Aged Cyp2e1 Knockout Mouse Model	UQCRC2	Reduced UQCRC2 protein expression, decreased Complex III activity	Abdelmegeed et al. (2016)
Non-alcoholic Fatty Liver Disease Rat Model	Complex III	Increased CIII protein expression	Ronis et al. (2013)

It is noteworthy that the tables in this review incorporate both cellular and animal models of NAFLD, necessitating a clear distinction between hepatocyte mitochondria and whole-liver mitochondria (Brenner et al., 2013). Hepatocytes, as the predominant parenchymal cell type in the liver, possess a high mitochondrial density to support their extensive metabolic demands, including glucose and lipid metabolism, protein biosynthesis, and detoxification. By contrast, assessments of whole-liver mitochondria unavoidably encompass mitochondrial populations derived from non-parenchymal cells, such as hepatic stellate cells, Kupffer cells, and liver sinusoidal endothelial cells, which differ from hepatocytes in mitochondrial abundance, morphology, and metabolic activity (Zhao et al., 2023). Accordingly, mitochondrial function measured in hepatocytes may not directly correspond to the averaged functional state of whole-liver mitochondria. This distinction is particularly critical in NAFLD models, as hepatocyte-specific mitochondrial alterations are key drivers of disease-associated metabolic dysfunction (Hunt et al., 2019).

#### Dysregulation of complex IV and V in energy supply

Mitochondrial complex IV, also known as cytochrome c oxidase, is the final enzyme in the mitochondrial respiratory chain (Kadenbach, 2021; Dietrich et al., 2024). Its primary function is to transfer electrons from cytochrome c to molecular oxygen (O2), generating water (H2O) (Brzezinski et al., 2021). Complex IV consists of 13 subunits. Among these, subunits I, II, and III are encoded by mitochondrial DNA (mtDNA) and form the catalytic center of complex IV. These three subunits are essential for electron transfer and proton pumping in the mitochondrial respiratory chain. The remaining 10 subunits are encoded by nuclear genes, synthesized in the cytoplasm, and then imported into the mitochondria (Brischigliaro and Zeviani, 2021). Mitochondrial complex V, also known as ATP synthase, is the last complex in the mitochondrial respiratory chain (Fernandez-Vizarra and Zeviani, 2021). Complex V is composed of 16 subunits, with these subunits encoded by both mitochondrial and nuclear genes. It consists of two main subunits: F1 and F0. The F1 subunit is located in the mitochondrial matrix, while the F0 subunit is embedded in the mitochondrial inner membrane (Singh, 2023). The main function of complex V is to catalyze ATP synthesis by utilizing the proton electrochemical gradient generated by the respiratory chain. It can also hydrolyze ATP through the reverse process (Del Dotto et al., 2024). Complex V synthesizes ATP from ADP, inorganic phosphate (Pi), and Mg2+, providing energy for the cell. In this process, complexes I, III, and IV transfer electrons while pumping protons from the mitochondrial matrix across the mitochondrial inner membrane into the intermembrane space, establishing the electrochemical gradient (Althaher and Alwahsh, 2023).

Electron leakage arises from reduced efficiency of terminal electron transfer at complex IV, leading to passive upstream electron escape and ROS generation, whereas electron overload results from functional constraints of complex V that induce a high membrane potential state, in which electron input exceeds ATP utilization capacity and triggers RET-dependent ROS bursts (Tabata Fukushima et al., 2024). Complex IV is the terminal electron acceptor of the mitochondrial respiratory chain, catalyzing the transfer of electrons from cytochrome c to molecular oxygen to form water, while simultaneously driving proton translocation across the membrane to maintain the membrane potential. In NAFLD, lipid overload can impair the assembly and stability of cytochrome c oxidase (COX), resulting in decreased complex activity. Lipid accumulation alters the lipid environment of the mitochondrial inner membrane, disrupting proper folding of COX subunits and supercomplex formation, thereby reducing electron transfer efficiency and ATP production while increasing ROS generation, ultimately promoting oxidative stress and hepatocellular injury (Gu et al., 2016; Durand et al., 2021). These protons are ultimately utilized by complex V to generate ATP. Therefore, complex V is a key enzyme in mitochondrial oxidative phosphorylation and chloroplast photophosphorylation for ATP synthesis (Cogliati et al., 2021). Complex V is responsible for synthesizing ATP using the proton motive force; however, under pathological conditions it may also operate in reverse, hydrolyzing ATP to maintain the membrane potential (Salewskij et al., 2020). In NAFLD models, mitochondrial damage and alterations in the proton gradient may increase ATP hydrolysis activity, thereby reducing energy efficiency and exacerbating metabolic stress. Moreover, impairment of complex V function can feed back to influence electron flow in upstream complexes, enhancing ROS production and further promoting lipid peroxidation and inflammatory responses (Nesci et al., 2015).

Most studies (Meng et al., 2024; Xu et al., 2022; Chimienti et al., 2021; Zhang X. et al., 2021; Zhao et al., 2018; Liu et al., 2015) focus on the overall complex IV (Table 4), observing a decrease in its expression and activity in NAFLD, which leads to weakened mitochondrial respiratory function, impaired mitochondrial function, and increased oxidative stress levels. Some studies have shown that in obese subjects, the activity of mitochondrial ETS complex IV is higher, with enhanced mitochondrial oxidative metabolism, and the increased oxidative metabolism in peripheral blood mononuclear cells (PBMCs) is associated with hepatic steatosis in obese young individuals (Shirakawa et al., 2023). Although the direct relationship between complex IV and NAFLD is not mentioned, hepatic steatosis is closely related to NAFLD, and the interaction between obesity and complex IV warrants further attention. Compared to visceral adipose tissue (VAT) in non-alcoholic fatty liver participants, individuals with non-alcoholic steatohepatitis (NASH) exhibit lower expression of oxidative phosphorylation complex IV (Pafili et al., 2022). In a high-fat diet-induced C57BL/6J mouse model of NAFLD, compared to normal mice, the expression of mitochondrial cytochrome c oxidase subunit IV (COX IV) in brown adipose tissue was increased, while its expression in white adipose tissue was decreased. This differential expression of complex IV subunits in adipose tissues deserves further investigation (Yoneshiro et al., 2018).

**TABLE 4:** Mitochondrial complexes IV and V dysfunction and their specific association with NAFLD.

Model	Mitochondrial complex IV and V target	Biological effect	References
High-fat Diet (HFD) Induced SD Rat Model; FFAs Induced HepG2 Cell Model (human)	Complex IV	Reduced COX IV protein and gene expression, mitochondrial dysfunction, lipid accumulation, steatosis, oxidative stress, and hepatocyte inflammation	Meng et al. (2024)
FFAs and TAA Induced Zebrafish and L02 Cell Models (human)	Overall Complex IV	Decreased COX IV activity, mitochondrial dysfunction, lipid accumulation	Xu et al. (2022)
High-fat and Fructose (HF-F) Diet Induced NAFLD Rat Model	Complex IV	Reduced COX-IV protein expression	Chimienti et al. (2021)
Oleic Acid (OA) Induced HepG2, L02 Cell Models (human)	Complex IV	Reduced COX IV protein expression	Zhang et al. (2021a)
High-fat Diet Induced Wistar Rat Model	Complex IV	Reduced mitochondrial complex IV protein expression	Zhao et al. (2018)
High-fat Diet and Streptozotocin Induced NAFLD Rat Model	Overall Complex IV	Decreased COX IV activity, reduced mitochondrial membrane potential	Liu et al. (2015)
Cholesterol-enriched High Saturated Fat Diet Induced Mouse Model	ATPA (Complex V)	Decreased ATPA protein expression, reduced Complex V protein levels, mitochondrial dysfunction	Lee et al. (2018)

Complex IV as a whole has been extensively studied, and most results show decreased complex IV activity in NAFLD models, leading to mitochondrial dysfunction, lipid accumulation, and increased ROS levels. One study targeting cytochrome c oxidase subunit IV isoform 1 (COX4I1) showed that increased COX4I1 activity leads to higher mitochondrial density and mass. Studies on complex V are relatively fewer (Table 4). One article (Murphy, 2009) showed that in the NAFLD model, the expression of subunit ATPA was reduced, and complex V activity decreased, triggering mitochondrial dysfunction. Overall, both complex IV and V likely undergo reduced activity and mitochondrial dysfunction under NAFLD conditions. Further research on complex V would enrich the understanding of the interaction between mitochondrial complexes and NAFLD, providing a more comprehensive reference for future studies.

### Mitochondrial complex regulators and other factors in NAFLD

#### PGC-1α (Peroxisome proliferator-activated receptor gamma coactivator 1-alpha)

PGC-1α is a key transcriptional coactivator of mitochondrial biogenesis and may promote the synthesis and assembly of respiratory chain complexes I–V by coordinating the expression of nuclear- and mitochondrial-encoded genes (Sukhorukov et al., 2024; Shi et al., 2024; Lou et al., 2024). Most existing studies are correlative, and the precise mechanisms by which PGC-1α and related factors specifically regulate the function and stability of complexes I–V remain to be elucidated. Studies have shown that Sanhuang decoction protects against obesity/diabetes-induced NAFLD in obesity and galr1 gene knockout mouse models by upregulating the PGC-1α/PEPCK signaling pathway (Fang et al., 2020). Further research has explored how Fenofibrate, through enhancing the PPARα/PGC-1α signaling pathway, promotes mitochondrial β-oxidation, enhances mitochondrial biogenesis and ATP production, and improves mitochondrial function to alleviate non-alcoholic fatty liver disease (NAFLD) (Wang et al., 2024). Under high-fat conditions, SIRT1 affects mitochondrial physiology and lipid autophagy through the PGC-1α pathway, thereby regulating various cellular characteristics (Jiang et al., 2021).

#### AMPK (AMP-activated protein kinase) and mitochondrial metabolism

AMPK is an energy-sensing kinase that is activated under conditions of cellular energy deficiency, such as a decreased ATP/AMP ratio. Most existing studies are correlative and suggest that AMPK may indirectly enhance the expression and assembly of respiratory chain complexes I–V by phosphorylating PGC-1α or its upstream regulators (Jiang, 2024). AMPK has been shown to induce mitochondrial fission by directly phosphorylating MFF (mitochondrial fission factor) during energy stress, and regulate mitochondrial autophagy through DRP1 (Cheng et al., 2024). After sustained energy stress, AMPK promotes mitochondrial biogenesis through transcriptional activation via PGC-1α, TFEB, or TFE3 (Garcia and Shaw, 2017). Dysfunction of respiratory chain complexes directly leads to an imbalance in mitochondrial fission, fusion, and selective mitophagy by altering mitochondrial energetic and redox states (Lacombe and Scorrano, 2024). Damage to complexes I–V restricts electron transfer, causes a decline or instability in membrane potential, and increases ROS production, thereby activating mitochondrial quality-sensing pathways centered on PINK1–Parkin to label and eliminate mitochondria harboring defective complexes; mitochondrial fission facilitates the segregation of damaged components, whereas fusion can partially buffer mild functional defects (Li et al., 2023; R et al., 2017). In contrast, when mitophagy is impaired, dysfunctional mitochondria cannot be efficiently removed, leading to the accumulation of defective respiratory chain complexes, exacerbated ROS generation, energy deficiency, and further amplification of dysfunction and pathological signaling (Shadel and Horvath, 2015). Studies have shown that royal jelly proteins (MRJPs) activate the AMPK/SIRT3 pathway *in vitro*, upregulating the expression of cytochrome c oxidase subunit IV, promoting energy metabolism and mitochondrial biogenesis, and alleviating NAFLD (Zhang et al., 2021a).

#### Mitochondrial supercomplexes

**FIGURE 1:**
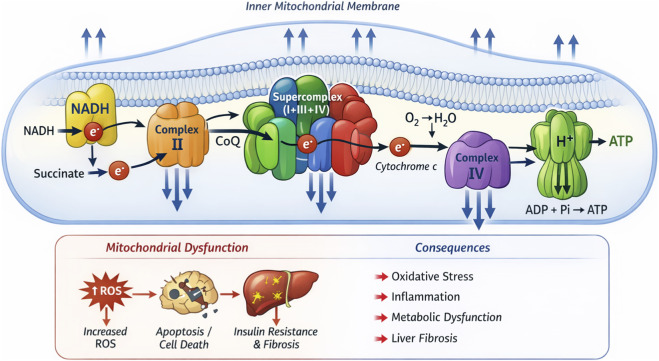
Mitochondrial Electron Transport Chain and Supercomplexes. The mitochondrial electron transport chain and the consequences of mitochondrial dysfunction. It depicts the interactions between Complexes I, II, III, IV, and V on the inner mitochondrial membrane. The diagram also shows how mitochondrial supercomplexes (aggregates of Complexes I, III, and IV) enhance the interactions between complexes, improving electron transfer efficiency and reducing ROS (reactive oxygen species) production. Below the diagram, the consequences of mitochondrial dysfunction are clearly illustrated. Excessive ROS production leads to cellular damage, apoptosis, insulin resistance, and liver fibrosis. Additionally, oxidative stress, inflammatory responses, and metabolic dysfunction are major adverse outcomes of mitochondrial dysfunction.

The mechanisms of the mitochondrial electron transport chain and mitochondrial dysfunction have been extensively documented in the literature (Vercellino and Sazanov, 2022). This review presents a schematic diagram that highlights the interactions between mitochondrial complexes and their effects on cellular energy production and overall health, while clearly illustrating the link between mitochondrial dysfunction and various diseases (See Figure 1). The arrangement of mitochondrial respiratory chain complexes into higher-order aggregates (supercomplexes) reduces ROS production by maintaining a high rate of electron transfer (Maranzana et al., 2013). In a cholesterol-fed NAFLD mouse model, the cholesterol load *in vivo* reduced the levels of Complex III and the assembly of respiratory supercomplexes (I1+III2+IV and I1+III2), leading to oxidative stress and liver damage (Solsona-Vilarrasa et al., 2019). The function of supercomplexes remains a topic of debate, but these superstructures may be related to the hypothesis of substrate oxidation efficiency and reduced ROS production in mitochondrial electron transport, making them potential therapeutic targets for NAFLD (Ramírez-Camacho et al., 2020). Most existing studies are primarily correlative analyses (Acin-Perez and Enriquez, 2014). In cardiac and renal mitochondria, ATP production primarily depends on long-chain fatty acid β-oxidation. Respiratory chain supercomplexes play a central role (Panov, 2024; Blaza et al., 2014): two large supercomplexes contain complex I, a dimer of complex III, and two dimers of complex IV, while a third smaller supercomplex contains a dimer of complex III and two dimers of complex IV. Fatty acid β-oxidation enzymes are physically associated with these supercomplexes, enabling efficient electron transfer. The generated QH_2_ can reverse electron flow through complex II and promote succinate formation, further enhancing β-oxidation. Oxidation of QH_2_ by the smaller supercomplex increases the NADH/NAD^+^ ratio and mitochondrial energy level, while transhydrogenase regulates NADP(H), ensuring ATP supply and energy homeostasis in the heart.

#### Mitochondrial Flashes

Mitochondrial Flashes are quantized signals within mitochondria that involve multiple changes, such as bursts of mitochondrial reactive oxygen species (ROS), transient alkalization of the matrix, and a temporary decline in membrane potential, occurring over a time scale of several tens of seconds (Feng et al., 2017). This phenomenon represents a novel form of fundamental mitochondrial activity, reflecting highly conserved mitochondrial functions. Mitochondrial Flashes are widely observed across multiple species and various cell types, manifesting whenever functional mitochondria are present (Hou et al., 2014).

Mitochondrial dysfunction plays a crucial role in the pathogenesis of NAFLD, contributing to oxidative stress, lipid accumulation, and hepatocyte apoptosis. Although Mitochondrial Flashes are indicative of mitochondrial activity, their direct implications in mitochondrial dysfunction, particularly in NAFLD, remain to be fully elucidated (Wang et al., 2012). The burst of superoxide anions associated with Mitochondrial Flashes could exacerbate oxidative stress, thereby influencing the progression of NAFLD. Additionally, these flashes may indicate an abnormal state of mitochondrial energy metabolism, linking them to metabolic dysfunction observed in NAFLD. Despite these associations, further research is needed to fully understand the specific relationship between Mitochondrial Flashes and NAFLD. Therefore, closely integrating mitochondrial flashes and advanced imaging technologies with the pathological context of NAFLD can enhance the biological interpretability of these emerging research areas.

## Discussion

Mitochondria are the primary organelles responsible for cellular energy production, generating ATP through oxidative phosphorylation (OXPHOS) to sustain normal hepatic metabolism and function (Deng et al., 2024). In metabolic disorders such as non-alcoholic fatty liver disease (NAFLD), mitochondrial dysfunction is recognized as a central mechanism underlying disease initiation and progression. Mitochondrial impairment leads to reduced electron transport efficiency, decreased ATP synthesis, and excessive reactive oxygen species (ROS) production, thereby triggering oxidative stress, inflammation, and apoptosis, which collectively drive NAFLD development (Di Ciaula et al., 2021). During energy metabolism, mitochondrial respiratory chain complexes I–V act in concert to mediate electron transfer and establish proton gradients essential for ATP generation (Prasun, 2020). Dysfunction of these complexes not only compromises OXPHOS efficiency but also disrupts lipid metabolism and activates cellular stress responses. Therefore, a systematic evaluation of functional alterations in complexes I–V is critical for elucidating their roles in NAFLD pathogenesis and progression. Accordingly, this review summarizes the reported changes in mitochondrial complexes I–V across different stages of NAFLD, as presented in Tables 1–4, and discusses their disease relevance in the preceding sections. We further propose that future studies should focus on the following characteristics of these complexes in NAFLD research:

First, most existing studies primarily emphasize overall mitochondrial activity or respiratory function, with relatively limited attention to the localization and functional roles of individual subunits within each respiratory chain complex. Current approaches largely rely on Western blot analyses that assess total complex expression, while specific subunits involved are often not clearly defined, and related reports remain scarce (Zhao et al., 2018; Xu et al., 2014). Therefore, future studies should explicitly identify and characterize the subunits under investigation to clarify their precise contributions to NAFLD initiation and progression, thereby strengthening the molecular basis for targeted interventions. Methodologically, mitochondrial subfractionation can provide preliminary localization of target subunits; co-immunoprecipitation and blue-native PAGE can be used to analyze subunit interactions and assembly states (Shi et al., 2021); high-throughput proteomics (e.g., LC-MS/MS) enables comprehensive profiling of subunit composition, expression changes, and post-translational modifications (Campolo et al., 2023); fluorescence labeling and immunofluorescence co-localization can verify intramitochondrial distribution (Agyemang et al., 2024); genetic manipulation using CRISPR/Cas9 or RNA interference allows functional validation by assessing effects on ATP production, ROS generation, and membrane potential (Tyumentseva et al., 2023); and cryo-electron microscopy offers high-resolution structural insights into subunit organization and interactions within the complexes (Yip et al., 2020).

Furthermore, Changes in mitochondrial complex activity or expression in NAFLD are inconsistent and warrant further investigation (Sangineto et al., 2021). In some models, electrons leak from Complexes I and III, increasing ROS production, while fatty acid oxidation enzymes CPT1A and CPT2 are upregulated, Complex II activity rises, and Complexes I, III, and V show varying decreases (Sangineto et al., 2021). It should be noted that mitochondrial reactive oxygen species (ROS) have a dual role: at moderate levels, ROS act as signaling molecules, regulating antioxidant gene expression, mitochondrial biogenesis, and quality control through pathways such as Nrf2, AMPK, or PGC-1α, thereby enhancing cellular adaptation to metabolic stress; however, excessive ROS that exceed the redox threshold or overwhelm antioxidant defenses can cause oxidative damage to proteins, lipids, and DNA, activate inflammatory and apoptotic signaling, and trigger pathological responses. Cell type also affects mitochondrial responses: FFA treatment reduces respiratory function in HepG2 cells but increases Complexes I and II activity in HepaRG cells (Maseko et al., 2024). Additionally, some clinical studies report enhanced mitochondrial respiration in NASH patients (Maseko et al., 2024). These findings suggest that mitochondrial complex function in NAFLD is influenced by factors such as cell type, disease stage, and metabolic context, highlighting the need to explore dynamic changes in each complex under different pathological conditions.

This study also found that most current research primarily focuses on changes in the overall mitochondrial function under different disease states, such as reduced oxygen consumption and weakened respiratory chain activity. However, studies on how the spatial structural changes of subunits within mitochondrial complexes and their assembly patterns affect mitochondrial function are still relatively scarce (Vercellino and Sazanov, 2022). For example, a recent study pointed out that under low-temperature conditions, Complex III of the mitochondrial respiratory chain undergoes a conformational shift of about 15°, and this spatial displacement enhances the electron transfer rate between Complexes I and III, thereby improving the overall mitochondrial function (Shin et al., 2024). This suggests that when investigating mitochondrial function changes during the onset and progression of NAFLD, the impact of complex conformational changes on functional status should not be overlooked. Additionally, some studies have shown that abnormal cholesterol accumulation can interfere with the assembly of mitochondrial supercomplexes. It was found that under cholesterol overload conditions, the dimerization of Complex III (CIII_2_) was significantly reduced, and the assembly of respiratory supercomplexes (e.g., CI_1_+CIII_2_+CIV and CI_1_+CIII_2_) was notably inhibited (Solsona-Vilarrasa et al., 2019). This structural remodeling may reduce the cooperative efficiency of the electron transport chain, further affecting ATP synthesis efficiency and mitochondrial homeostasis. Therefore, in future NAFLD research, in addition to focusing on the overall activity changes of the complexes, greater attention should be given to subunit conformational changes, spatial coordination between complexes, and the dynamic regulation of supercomplex assembly, in order to gain a more comprehensive understanding of the role of mitochondria in the disease process.

Then, studies have shown that researchers focus on the endogenous regulators of mitochondrial complexes (Cicuéndez et al., 2025), which are molecules produced within the cell—such as transmembrane proteins, enzymes, or signaling factors—that modulate mitochondrial functions like electron transport, redox balance, and apoptosis regulation without external stimulation. For example, MCJ is a transmembrane protein located in the mitochondrial inner membrane (encoded by the nuclear gene Dnajc15), and the deletion of MCJ leads to an increase in Complex I activity (Barbier-Torres et al., 2020). In addition, the excessive production of reactive oxygen species (ROS) triggered by mitochondrial stress may inhibit insulin receptor substrates (IRS) and their downstream signaling pathways through phosphorylation, thereby promoting the development of insulin resistance (Masenga et al., 2023; Ayer et al., 2022). Meanwhile, oxidative stress and inflammation caused by mitochondrial dysfunction are closely linked to hepatic fibrosis (Hammerich and Tacke, 2023). The overproduction of ROS can activate the NLRP3 inflammasome and other related inflammatory pathways, which in turn induces the activation of hepatic stellate cells (HSCs) and promotes the progression of liver fibrosis (Yang et al., 2014). Moreover, mitochondrial damage can alter cellular metabolic patterns, leading to hepatocyte apoptosis or necrosis, further exacerbating the occurrence of liver fibrosis (Yang et al., 2014). Therefore, mitochondrial dysfunction plays a crucial bridging role between insulin resistance and hepatic fibrosis, with both processes influencing each other and collectively driving the progression of NAFLD.

Lastly, advanced technologies are required to enable in-depth investigation of the structure and function of mitochondrial complexes. Although mitochondria contain approximately 1,000–2,000 proteins—among which respiratory chain proteins are the most critical—their extremely small volume presents substantial technical challenges for structural studies (Pfanner et al., 2019). Traditional X-ray crystallography is suitable for relatively simple proteins but is limited in resolving large, dynamic assemblies such as mitochondrial complex I and its supercomplexes because of their high molecular weight and structural flexibility (García-Nafría and Tate, 2021). Single-particle cryo-electron microscopy (cryo-EM) has enabled high-resolution three-dimensional reconstruction of mitochondrial complexes, providing key insights into their composition and organization; however, it generally focuses on a limited number of proteins at a time and relies on purification steps that may disrupt native structures or functions (Yip et al., 2020). To overcome these limitations, *in situ* cryo-EM has emerged as a powerful approach, allowing biomolecules to be visualized within cells or tissues in a near-physiological state (Hou and Zhang, 2025; Waltz et al., 2025). Using this technique, the structure of the TIM22 complex has been resolved at 3.8 Å resolution, with clear identification of seven subunits (Zhang et al., 2021b), and temperature-dependent structural remodeling of respiratory chain supercomplexes in brown adipocytes has been revealed, advancing our understanding of cold adaptation mechanisms (Shin et al., 2024; Chen et al., 2019). Moreover, recent *in situ* cryo-EM studies have shown that lipid-mediated “water chains” participate in proton transfer, highlighting that full mitochondrial complex function depends on the integrated molecular environment (Zheng et al., 2024).

From a therapeutic perspective, accumulating evidence converges on a multilevel regulatory strategy centered on mitochondrial respiratory chain complex I. As the primary entry point for electron transfer and a major source of mitochondrial reactive oxygen species (ROS), dysfunction of complex I is a critical driver of energy metabolic imbalance and oxidative stress (Dan Dunn et al., 2015). Accordingly, fine-tuning complex I activity through pharmacological or nutritional interventions—rather than simple inhibition or activation—may help restore the NAD^+^/NADH balance, reduce excessive ROS production, and thereby enhance overall mitochondrial bioenergetic efficiency (Zorov et al., 2014). Building on this concept, targeting complex I–associated reverse electron transport (RET) provides a more precise approach to alleviating pathological oxidative stress. Under pathological conditions characterized by high membrane potential and excessive electron supply, RET-driven ROS generation is considered a major source of oxidative damage; selective suppression of aberrant RET, while preserving normal forward electron transport, may effectively mitigate oxidative stress without compromising ATP synthesis (Onukwufor et al., 2019; Robb et al., 2018). Finally, restoring the stability of respiratory chain supercomplexes at the structural level may represent a key integrative step in these functional interventions, as supercomplex disassembly impairs electron transfer efficiency and exacerbates electron leakage and ROS production (Guan et al., 2022; Kohler et al., 2023). Strategies aimed at maintaining or re-establishing supercomplex integrity—through modulation of membrane lipid composition, assembly factors, or small molecules that stabilize protein–protein interactions—could synergistically improve respiratory efficiency, reduce oxidative damage, and enhance mitochondrial stability and adaptability under pathological conditions.

In summary, the structure and function of mitochondrial complexes have significant impacts on diseases, particularly in metabolic diseases like NAFLD, which have garnered increasing attention. Although current research largely focuses on changes in overall activity, the specific structure and assembly state of individual subunits have not been fully explored. Future studies should integrate advanced high-resolution techniques, such as *in situ* cryo-electron microscopy, to further reveal the spatial conformation and dynamic changes of mitochondrial complexes under both physiological and pathological conditions. Additionally, the regulatory mechanisms of lipid environments on complex function also warrant further investigation. These studies will provide a solid foundation for elucidating the mechanisms of mitochondrial dysfunction, discovering precise therapeutic targets, and advancing the diagnosis and treatment of NAFLD and related diseases.

## Glossary

ATP: Adenosine triphosphate
BN-PAGE: Blue native polyacrylamide gel electrophoresis
CDAHFD: Choline-deficient, L-amino acid-defined, high-fat diet
CI: Complex I—NADH:ubiquinone oxidoreductase
CII: Complex II—Succinate dehydrogenase
CIII: Complex III—Ubiquinol:cytochrome c oxidoreductase
CIV: Complex IV—Cytochrome c oxidase
Co-IP: Co-immunoprecipitation
CPT1A: Carnitine palmitoyltransferase 1A
CPT2: Carnitine palmitoyltransferase 2
CRISPR/Cas9: Clustered regularly interspaced short palindromic repeats/CRISPR-associated protein 9
Cryo-EM: Cryogenic electron microscopy
Cryo-ET: Cryogenic Electron Tomography
CYP2E1: Cytochrome P450 2E1
DHT: Dihydrotestosterone
ERRα: Estrogen-related receptor alpha
FFA/FFAs: Free fatty acids
HepG2: Human hepatocellular carcinoma cell line
HFD: High-fat diet
LC-MS/MS: Liquid chromatography-tandem mass spectrometry
MCJ: Mitochondrial carboxyl-Jase
NAFLD: Non-alcoholic fatty liver disease
NRF1: Nuclear respiratory factor 1
PEPCK: Phosphoenolpyruvate carboxykinase
PFA: Paraformaldehyde
PGC-1α: Peroxisome proliferator-activated receptor gamma coactivator 1 alpha
RCS: Respiratory chain supercomplex
SDHA: Succinate dehydrogenase complex, flavoprotein subunit
SDHB: Succinate dehydrogenase complex, iron-sulfur subunit
SIRT1: Sirtuin 1
SIRT3: Sirtuin 3
TAA: Thioacetamide
Tfe3: Transcription factor E3
Tfeb: Transcription factor EB
TIM22: Translocase of inner mitochondrial membrane 22
UQCRC2: Ubiquinol-cytochrome c reductase complex core protein 2
X-ray crystallography: X-ray diffraction for crystallized structures
