# Supplementary figures and images for: Roles of mitochondrial complexes in non-alcoholic fatty liver disease
Source: Front Mol Biosci. 2026 Feb 23;13:1752024. doi: 10.3389/fmolb.2026.1752024 (PMC12968024; doi:10.3389/fmolb.2026.1752024)

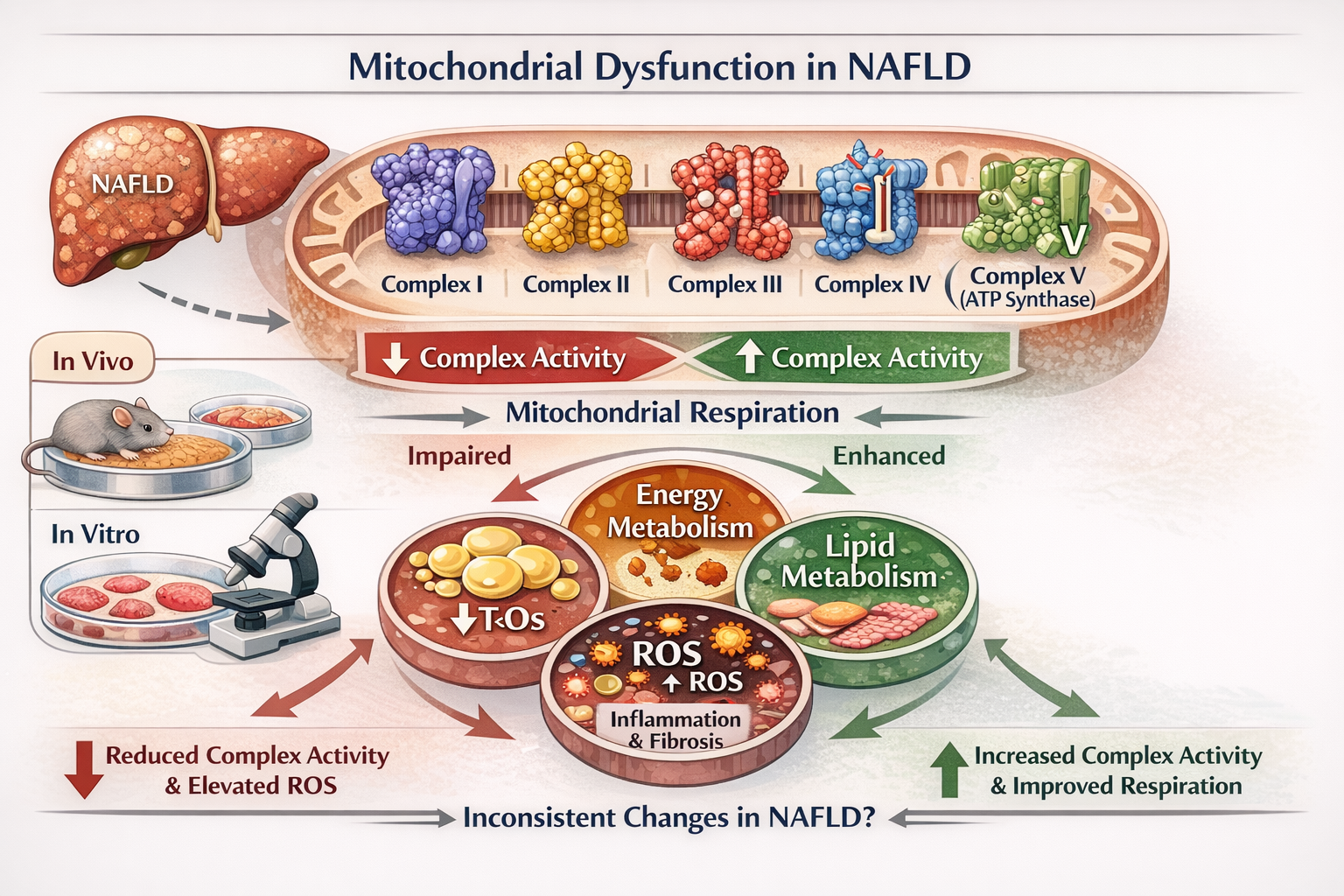

Supplement: Supplementary file 1 [file Image2.png]

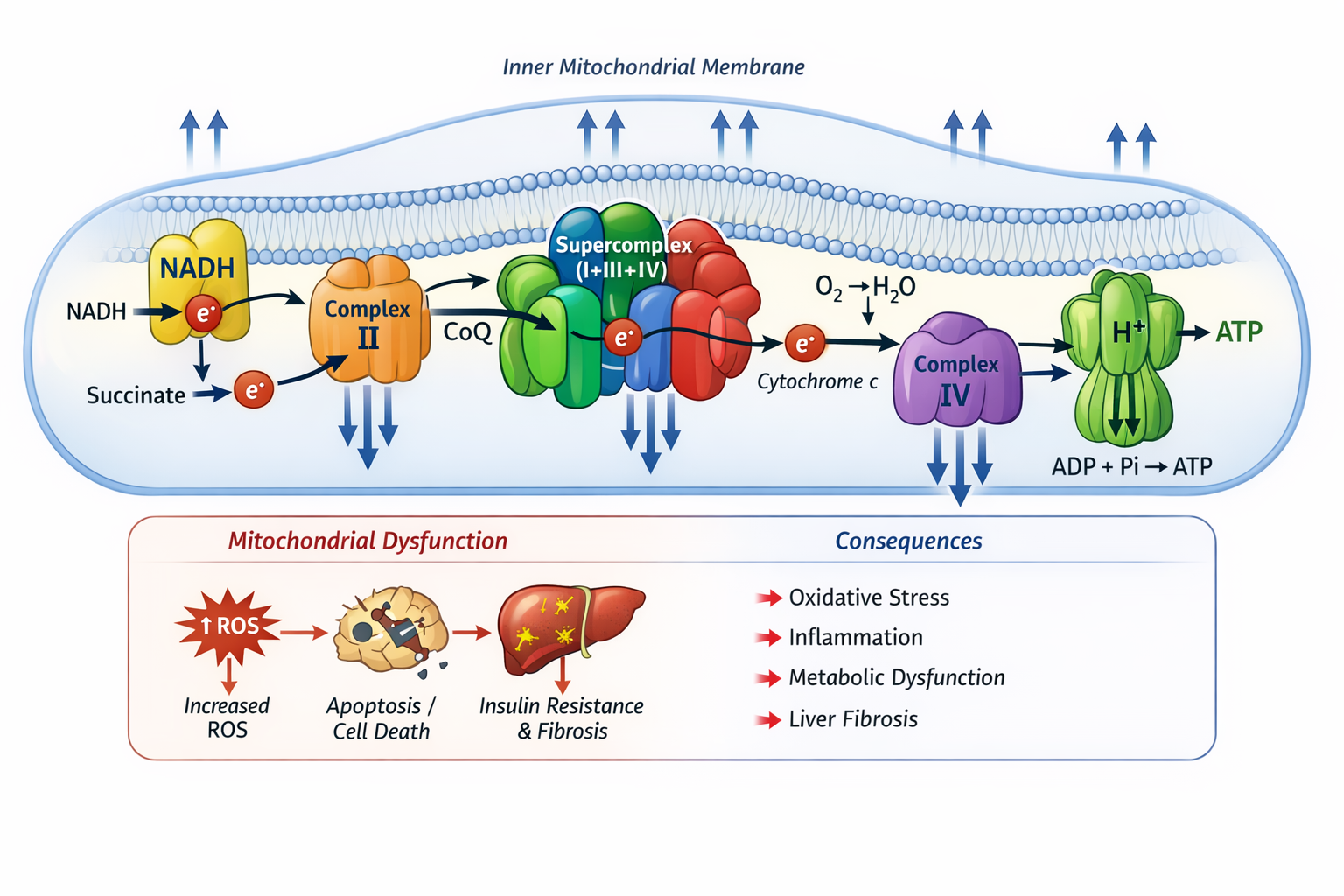

Supplement: Supplementary file 2 [file Image1.png]
